# Journals Operating Predatory Practices Are Systematically Eroding the Science Ethos: A *Gate and Code* Strategy to Minimise Their Operating Space and Restore Research Best Practice

**DOI:** 10.1111/1751-7915.70180

**Published:** 2025-06-19

**Authors:** Kenneth Timmis, Paul Williams, Zeynep Ceren Karahan, Purificación López‐García, Paul Rainey, Max Chavarria, Chris Greening, Karen Steward, John E. Hallsworth, Cristina Silva Pereira, Rafael Giraldo, Willy Verstraete, Stipan Jonjić, Juan Luis Ramos, Olga Nunes, Antonio Ventosa, Rachel Armstrong, Angela Sessitsch, Eliora Ron, Hui Wang

**Affiliations:** ^1^ Institute of Microbiology Technical University Braunschweig Braunschweig Germany; ^2^ Biodiscovery Institute and School of Life Sciences University of Nottingham Nottingham UK; ^3^ Department of Medical Microbiology and Ibn‐i Sina Hospital Central Microbiology Laboratory Ankara University School of Medicine Ankara Turkey; ^4^ Ecologie Systématique Evolution, CNRS Université Paris‐Saclay, AgroParisTech Gif‐sur‐Yvette France; ^5^ Department of Microbial Population Biology Max Planck Institute for Evolutionary Biology Plön Germany; ^6^ Escuela de Química & CIPRONA Universidad de Costa Rica San José Costa Rica; ^7^ Centro Nacional de Innovaciones Biotecnológicas (CENIBiot), CeNAT‐CONARE San José Costa Rica; ^8^ Department of Microbiology, Biomedicine Discovery Institute Monash University Clayton Australia; ^9^ Technology Networks Sudbury UK; ^10^ Institute for Global Food Security, School of Biological Sciences, Medical Biology Centre Queen's University Belfast Belfast UK; ^11^ Instituto de Tecnologia Química e Biológica António Xavier Oeiras Portugal; ^12^ Department of Microbial Biotechnology National Centre for Biotechnology (CNB‐CSIC) Madrid Spain; ^13^ Center for Microbial Ecology and Technology (CMET) Ghent University Ghent Belgium; ^14^ Center for Proteomics, Faculty of Medicine University of Rijeka Rijeka Croatia; ^15^ Consejo Superior de Investigaciones Científicas Estación Experimental del Zaidín Granada Spain; ^16^ LEPABE‐Laboratory for Process Engineering, Environment, Biotechnology and Energy, Faculty of Engineering University of Porto Porto Portugal; ^17^ Department of Microbiology and Parasitology, Faculty of Pharmacy University of Sevilla Sevilla Spain; ^18^ Department of Architecture KU Leuven Ghent Belgium; ^19^ Bioresources Unit AIT Austrian Institute of Technology Vienna Austria; ^20^ The Shmunis School of Biomedicine and Cancer Research Tel Aviv University Tel Aviv Israel; ^21^ Department of Environmental Science and Engineering Tsinghua University Beijing China

**Keywords:** journal accreditation, journal and editorial code of conduct, peer review, predatory practices in research publication, publication inflation, science ethos, science management, special issues

## Abstract

Scientific research seeks to extend knowledge and understanding, an activity that perhaps more than any other advances society and humanity. In essence, it is the search for truth. But, because it seeks new knowledge, there is little or no benchmark for appraisal of the plausibility or validity of the immediate conclusions drawn from new information gained, no instant confirmation. For this and other reasons, the science ethos requires the highest level of *rigour* to ensure the highest level of probability that new findings are true, or at least the most plausible under the prevailing circumstances and state of knowledge. Research is only as good as its degree of *rigour*. Rigour comes through intensive and comprehensive scientific training and mentoring that teaches critical and agnostic evaluation of new results, self‐scrutiny and self‐criticism. Additional rigour comes via independent scrutiny and validation: peer review of results and interpretations submitted as publications, and peer repetition of key experiments. However, the current proliferation of publication vehicles whose business model is based on maximisation of papers published, and the revenue stream of article processing charges (APCs) they generate, is promoting an insidious degradation of rigour and quality standards of reviewing–editing practices. Such *predatory practices* result in the systematic degradation of research quality and its “truthfulness”. Moreover, they undermine the science ethos and threaten to create a new generation of scientists that lack this ethos. These trends will inevitably progressively erode public trust in scientists and the research ecosystem. This Editorial is a call for action to all actors, in particular leaders, in scientific research to oppose predatory practices in science dissemination—to restrict the operational space of those responsible for such practices—in order to restore and maintain research rigour and the science ethos and to prevent a downward spiral of research quality. It proposes two linked actionable solutions to the problem, one for the “pull” element of predatory practices and one for the “push” element of research ecosystem management practices, especially those promoting the *publish or perish* mentality, that drive authors to publish in journals with predatory practices. To counter the “pull”, we propose a solution based on the principle of *prevention, rather than cure,* and list a number of essential policy decisions and actions that should be taken at all levels of the science chain/cloud to achieve this. A central plank of the concept is *journal accreditation*, without which a journal would be ineligible for payment of APCs from public funds. For accreditation, a journal would need to convincingly demonstrate adoption of a prescribed *journal code of conduct*. Ideally, accreditation would also be required for inclusion in journal indexing and ranking services and bibliographic databases. To counter the “push”, we propose a top‐down imposition of a cultural change in science management that ensures merit‐based success of scientists and their careers, research best practice, improved education and mentoring of younger scientists in the science ethos and greater support of them in their careers. This must include explicit recognition of the crucial role of peer reviewing for the good health of the research enterprise, its incentivisation and appropriate appreciation of the time and effort involved. To orchestrate this change, we propose the creation of a multi‐stakeholder alliance whose brief is to develop the framework and implementation strategy for changes in the research ecosystem. This Editorial also exhorts all actors to embrace the principle of *publish less, publish better* and to use public funding provided by tax revenues more effectively to perpetually raise the bar of science quality, dissemination and potential to advance humanity.

## 1 The Importance and Value of Science

Scientific research is the pursuit of new knowledge and understanding, which is central to the advancement of humanity and the solution or mitigation of its problems (e.g., see Basavanthappa 2024). It tells us who we and other organisms of the biosphere are, how our planetary home functions and how we interact with it and the other creatures it houses. It enables us to determine the factors that influence our well‐being, the biosphere and the planet and to develop and implement strategies and actions to overcome the threats they face. The value of scientific research to society is incalculable: just consider advances in medicine and agriculture that over a relatively short period of time have enabled a huge improvement in the average quality of life and a doubling of human longevity, in communication and the digital world, in knowledge of how things work and the commercial applications resulting from this knowledge, etc., a fact that many people and governments recognise and appreciate by investing significant tax revenues in research (e.g., Lepori et al. 2023; Cologna et al. 2025).

Science is the pursuit of truth or, at least, the most likely answer to a question/explanation of a phenomenon (*How does something work? Why/how do we get sick? How can we make a sick person better? How can we prevent the planet getting warmer? How can we feed more people? Etc?*). Knowledge and truth, as opposed to belief, dogma and propaganda, are agnostic: they are unbiased, do not take sides, are not part of an agenda, do not try to please. Crucially, the pursuit of truth necessitates rigour, because poorly conducted science can result in misleading conclusions/truths, sometimes leading to policies and actions that can engender serious harm, for example, by promoting vaccine or climate scepticism (https://theconversation.com/scientific‐certainty‐survival‐kit‐how‐to‐push‐back‐against‐skeptics‐who‐exploit‐uncertainty‐for‐political‐gain‐173578), (https://time.com/5175704/andrew‐wakefield‐vaccine‐autism/; https://www.unicef.org/montenegro/en/stories/mmr‐vaccine‐does‐not‐cause‐autism; https://www.who.int/news‐room/spotlight/ten‐threats‐to‐global‐health‐in‐2019; Larson 2022) (https://community.wmo.int/en/world‐climate‐programme‐wcp; https://geosci.uchicago.edu/~archer/warming_papers/charney.1979.report.pdf; https://www.weforum.org/stories/2020/01/climate‐science‐global‐warming‐most‐sceptics‐country/; Hornsey et al. 2018), or inadequate responses to pandemic or climate crises (Wise 2021; https://www.scientificamerican.com/article/how‐the‐u‐s‐pandemic‐response‐went‐wrong‐and‐what‐went‐right‐during‐a‐year‐of‐covid/; https://www.who.int/emergencies/disease‐outbreak‐news/item/2025‐DON561; https://www.cdc.gov/measles/data‐research/index.html; https://dshs.texas.gov/measles; https://energyinnovation.org/wp‐content/uploads/Cost_of_Delay.pdf).

However, the character qualities of scientists are no different from those of other career groups, collectively exhibiting a broad range of shades of integrity, creativity, precision, bias and self‐interest. Thus, in order to counter behaviours deviating from those essential to truth finding, a comprehensive quality control chain has evolved.

## 2 The Quality Control Chain in Research

*Research training is intensive and involves mentoring to embed rigour*. Becoming a researcher requires a long and intensive training in academia, almost always involving study for a Bachelor's Degree, often also a Master's Degree and, for the majority, also a doctorate, a Ph.D. or an M.D., a training that altogether typically lasts between 6–10 or 10–12 years, respectively. During this period, students are trained in the principles and practice of research. To qualify as an independent researcher usually requires a further postdoctoral training of typically 2–4 years during which the scientist is actively mentored by an experienced head of a research group and its more senior members. This decade or more of training (most academics justly maintain that learning and training are indeed lifelong) is not only intended to provide the skills of research, and define the theoretical framework, barriers and operating space of science, but also the spirit of research—the single‐minded search for truth with honesty and integrity; *the science ethos*—and to induct the new researcher into the community of scientists whose work is guided strictly by the scientific *code of conduct* (see, e.g., ‘The European Code of Conduct for Research Integrity’ https://allea.org/wp‐content/uploads/2023/06/European‐Code‐of‐Conduct‐Revised‐Edition‐2023.pdf). At any point during the training, the trainee can be ejected from (during formal studies and training), or strongly discouraged from continuing (thereafter), the research career path, if the science ethos is found to be wanting.

*Science training is not similar to the training in other fields of endeavour*: ‘Standing on the shoulders of giants’. While researcher training within the science community may superficially seem to be the same as the apprentice system within professional guilds, and the learning and training throughout a career that is normal for many/most professions, there is a key difference. Once a plumber, who has also received long and detailed training in plumbing and quality control, installs a tap, the exercise has reached an end. If the tap is installed badly, it will not prejudice the installation of a tap somewhere else by another plumber. In contrast, science builds continuously on previous advances (hence the famous phrase: *standing on the shoulders of giants* by Bernard of Chartres and Isaac Newton (https://en.wikipedia.org/wiki/Standing_on_the_shoulders_of_giants)): new ideas, concepts and hypotheses that significantly advance science evolve from the cloud of knowledge, understanding and expertise existing at that moment and, in particular, upon new information, methods and concepts that have just been published. A badly conducted experiment by someone somewhere that produces results that are not trustworthy, and leads to incorrect conclusions, can cause another scientist elsewhere to fail by designing an experiment badly or conducting a useless one. This may not only involve significant and unnecessary wastage of resources, which include time (which is often measured in years), labour (usually of multiple researchers and support staff), materials, infrastructure and the taxpayer funding that covers their costs, but, in some cases, also failure of scientists involved to gain essential qualifications and/or loss of livelihood due to an inability to secure an extension of funding. If the poor result is of importance to many scientists, the impact and wastage are amplified.

*Experimental design/research design*. To ensure best quality science, with emphasis on rigour, reproducibility, agnostic interpretation of results and insensitivity to outside pressures for a ‘desired’ result/conclusion (e.g., for commercial, political or career advancement reasons), there is a strict procedure for initiating a research project that usually involves *inter alia*:

- reading everything feasible that is known about the subject of interest,
- interrogating available information to identify the primary bottleneck restricting advancement of knowledge. In some cases, there may not be sufficient information available, so it is necessary to “explore”, to become a “naturalist”, like Charles Darwin (https://www.rmg.co.uk/stories/topics/charles‐darwin‐one‐britains‐most‐celebrated‐naturalists), in order to discern a good way forward,
- formulating a pertinent question, the answer to which can provide new insights,
- formulating a testable hypothesis whose validation or refutal will advance our understanding,
- designing a rigorous experiment/series of experiments/procedures that will provide the required information/answer and, crucially, that is reproducible (e.g., Casadevall and Fang),
- where possible, designing studies to enable valid statistical assessment of the reproducibility, in order to provide a quantified measure of the confidence of the results obtained, and the conclusions that can be drawn from them (although it is important to note that statistical significance does not always equate with biological significance).

*The renowned and valued academic freedom relates to the choice of study object, the idea to be tested, the experiment to be carried out and some working practices, such as the hours of the day/days of the week worked. However, the manner in which experiments are conducted is highly restricted: in this fundamental element of the scientific endeavour, there is little operating space/freedom of choice*.

*Self‐ and peer‐scrutiny*. Scientific research is also special because its product—the results and conclusions—is usually made promptly available to other researchers through presentation in internal and external seminars and conferences, and publication in research journals; that is, it is subject to an exceptional degree of repeated critical and rigorous scrutiny and quality control. This is not the same as the usual, internal, quality control of an industrial product, nor web‐based public ranking of products that may be subject to a range of different biases, not least the recommendations of ‘influencers’. Research reports are subject to a multi‐stage quality assessment which involves:

- author responsibility to make a fair and exhaustive search for all possible explanatory causes of results obtained, through objective consideration and discussion with others of alternative explanations,
- author responsibility at the report writing stage to compare the new results and integrate with, and benchmark against, relevant existing knowledge,
- peer review of the report submitted to a journal by independent experts who are usually anonymous and provide criticisms and suggestions for improvement. Many reports are initially rejected on the basis of such criticisms but, if the improvement recommendations are implemented, may subsequently be published by the journal initially selected or another,
- critical scrutiny and assessment of the validity and significance of results in the improved paper by the research community at large following its publication,
- peer repetition, confirmation and validation of the experiments central to important conclusions, as a vital prelude to their own exploitation of the new knowledge to advance knowledge further,
- over time, peer consideration of the results and conclusions in the context of new advances, and the perpetual raising of the quality bar.

Nullius in verba: *scepticism as a key element of the science ethos and further restriction on the scientist's operating space*. While trust plays an important role in many spheres of activity—such as healthcare, religion, building and plumbing, politics, diplomacy, law—and public ranking of practitioners in such services is often directly related to the levels of trust they generate—a central plank of the research process is *organised scepticism* (Merton 1942), or *Nullius in verba*—“take nobody's word for it”—the motto of the Royal Society (https://royalsociety.org/about‐us/who‐we‐are/history/), displayed on its coat of arms (https://commons.wikimedia.org/wiki/File:Coat_of_arms_of_the_Royal_Society.svg), as the default attitude to new results that are vital to one's own research until they are independently verified. Scientists trust the science ethos, not necessarily the results and interpretations of their peers. Scepticism is ingrained in the research mentality: even within a research group practicing experimental (‘wet’) science, individuals may insist on making and using their own reagents because they may not be fully confident of those of their colleagues. This obviously engenders duplication of effort and avoidable additional costs, but may be considered to be essential to avoid the possibility of even greater wastage of time, effort and costs of failure, due to use of a reagent whose preparation, use and storage might not have been subject to one's own standards. The improper handling of reagents is promoted by widespread lack of awareness of the purpose their individual components serve and further exacerbated by the use of commercial “kits” containing all components needed for an experiment (the equivalent of “packet mixes” for cakes and other food dishes: if a desired result is not achieved, it is not possible to optimise by rationally varying individual ingredients).

**Scepticism and its value**

Scepticism of published results and their interpretation is the scientist's armour against poor science, personal and collective harm through wasted time, effort, resources and precious research funds, and reputational damage.

*The responsibility of scientists to maintain* the *highest standards and the scientific ethos*. The agnostic search for truth and scepticism of one's new discoveries and those of others until verified are key reasons why the public trusts scientists and the new knowledge revealed by their work. It is this trust that persuades generations of governments to fund scientific research through tax revenues (Lepori et al. 2023). In return, it is expected that scientists respect the science ethos, act with integrity in a trustful manner and single‐mindedly seek the truth in order to advance knowledge, some of which will lead to applications that directly benefit society.

**Researchers carry a significant burden of responsibility vis‐a‐vis**

- their peers, who can waste much time, intellectual effort and resources by basing new work on incorrect methodology, results and conclusions (see, e.g., Brodeur et al. 2024),
- their students and mentees, who propagate the ethics and practices they absorb in training,
- their institutions, whose reputations are built on the work carried out by the research teams they recruit, house and support,
- themselves, because the reputations they build are based upon the quality of their published work,
- the general public, who fund through taxes much of the academic research carried out and who deserve both a high level of quality control and a return on investment, for example, through advances in healthcare, etc. (Timmis and Hallsworth 2024),
- the legal system, which calls upon scientific knowledge to support/refute/clarify proposals/assumptions/evidence,
- the body politic, which relies on the latest available research to develop policy (Surkovic and Vigar 2022), particularly in relation to urgent needs—witness the COVID‐19 pandemic (Ball 2021; https://www.oecd.org/content/dam/oecd/en/publications/reports/2023/07/covid‐19‐and‐policy‐for‐science_263dd52f/8f86e60b‐en.pdf; https://www.oecd.org/content/dam/oecd/en/publications/reports/2023/07/covid‐19‐and‐science‐for‐policy‐and‐society_af405289/0afa04e2‐en.pdf) and global warming https://wmo.int/media/magazine‐article/critical‐role‐of‐observations‐informing‐climate‐science‐assessment‐and‐policy; https://unfccc.int/topics/science/the‐big‐picture/science‐in‐the‐unfccc‐negotiations—but also in relation to the development of a knowledge‐based economy, such as the bioeconomy (https://www.naturefinance.net/wp‐content/uploads/2024/05/ENG‐TheGlobalBioeconomy_FINAL.pdf), Green Deal (https://europeanmovement.eu/policy/delivering‐a‐strong‐european‐green‐deal/), and so forth.

*The need for collaboration and consequences of reputational damage*. Science is a largely collaborative enterprise (Camacho et al. 2024), involving different groups, some of which work together and others of which share/exchange vital, sometimes unique reagents, materials, equipment, methods and ideas (intellectual property). Given that scientists are by nature sceptical, an important factor that promotes and cements collaborations is *reputation*—for rigour, quality, originality, dependability. Loss of reputation can be highly damaging to the ability to collaborate freely and hence progress at a speed that is competitive. Reputational damage not only compromises collaboration but also mentoring, the ability to obtain funds vital to research and, crucially, public trust. Avoidance of reputational damage is vital to a successful research career, a powerful inducement of integrity in science and a major constraint on the operational space of scientist behaviour.

*Peer review: the vital gatekeeper in the quality control chain and maintainer of minimal operating space for dishonest science*. An essential product of most academic research is the publication of new results and their conclusions. Publication usually occurs in research journals, most of which are produced by for‐profit publishing enterprises that charge a fee for each paper published, an Article Processing Charge or APC, or a centralised (e.g., institutional library) or personal subscription for the entire journal (https://www.theguardian.com/science/2017/jun/27/profitable‐business‐scientific‐publishing‐bad‐for‐science; Trueblood et al. 2025). Traditionally, journals have Editors who are recognised experts in the relevant field of research and who steer the editorial process. While Editors may make an accept (usually exceptionally) or decline decision upon receipt and initial assessment of the submission (so‐called *desktop decision*), they often submit the paper to critical review by 2–3 experts in the field and make decisions based on the reviews and recommendations received. Editorial rejection of submitted manuscripts resulting from reviewer recommendations is a vital element in the gatekeeping process that maintains scientific standards and reduces operating space for sloppy, poor and dishonest science. Rejection can be definitive or conditional, in which case revisions such as new experiments, analyses, or interpretations may be required in order to be considered further for publication. Peer review is the vital independent evaluation of a scientific work and the primary barrier to poor science and scientific mis‐ and dis‐information (Anderson and Ledford 2021; Steer and Ernst 2021). It is also the main bulwark against dishonest science, a bulwark against loss of public confidence in science and its agnostic nature. *Peer review is a central plank of research quality control in science*.

*Peer review is responsible for huge improvements in submitted and eventually published research papers*. While the above focuses on the gatekeeping function of peer review resulting from reviewer recommendations, the output of reading, digesting and commenting on a piece of research and the way it is presented in a submitted manuscript are critical expert reviews: detailed texts that usually constitute a constructive and supportive road map for improvement of the work and its reporting, a road map that usually enables significant, and in some cases exceptional (e.g., in cases where important implications of findings are not apparent to the authors), betterment of the manuscript (see, e.g., Kelly et al. 2014).

*Rigorous peer review takes time and is usually incompatible with rapid editorial processing*. Reviewing ability is a skill/quality that is progressively learned over time, involving personal experience of the treatment of one's own manuscript submissions, mentoring, *ad hoc* reviewing, becoming a member of an editorial board and, finally, becoming an editor. The experience gained during the processes of mentoring and manuscript submission‐reviewing‐editing—learning what to look for in a submission, what to propose to correct weaknesses, how to support authors and help them improve their manuscript—is vital to the maintenance of science quality (e.g., see Casadevall et al. 2024). Rigorous reviewing of multi−/inter‐disciplinary research is particularly challenging because it often requires interdisciplinary experts, who are generally not abundant and thus in high demand (Alberts et al. 2008). Crucially, careful reviewing of new research requires dedication of considerable time in the lives of already overburdened scientists at all career stages. Thus, rigorous peer review of research papers (Editorials, Opinion papers and the like, which express personal views, excepted) is not ordinarily compatible with rapid editorial processing.

## 3 Current Danger of Predatory Publication Practices: Insidious Degradation of the Quality Control Chain and Erosion of the Science Ethos

**Use of the term predatory in this discourse**

The term predatory practice in the context of the dissemination of research findings has been used in different ways with different meanings. In this paper, journals employing predatory practices are considered to be exploitative academic publications that charge authors fees without providing legitimate peer review, adequate editorial oversight or rigorous quality control. They often prioritise profit over scholarly integrity, misleading researchers by mimicking aspects of reputable journals while publishing low‐quality or unverified research.

*Editorial rejection and demands for extensive revision of submitted manuscripts are diametrically opposed to the business model of journals characterised by predatory practices*. The research and publishing worlds are currently faced by an unprecedented explosion in journals employing predatory practices: journals that prioritise profit over quality, in some cases with little or no concern for science, its need for rigour and best practice, and especially its responsibility to society and taxpayers who fund much of its work (Beall and DuBois 2016; Cobey et al. 2018; Frandsen 2019; Amsen 2024; Hanson et al. 2024; Parker et al. 2024; Trueblood et al. 2025). The goal is maximisation of articles published and the revenue stream of APCs they engender. The common practice is, unlike traditional journals practicing rigorous peer review that often leads to initial rejection of a submitted report, to accept almost all submitted fee‐paying papers rapidly, either with only cursory review (and even self‐review, see Ferguson et al. 2014), or by ignoring reviewer requests for further experimental support of conclusions. Conditional or definitive rejection of the submission is unlikely. The practice of rapid editorial throughput and reluctance to demand rigorous review by known experts is undoubtedly facilitating the increase in fake reviews, sometimes from so‐called “review mills” (e.g., see Haug 2015; Oviedo‐García 2024).

**FIGURE 1:**
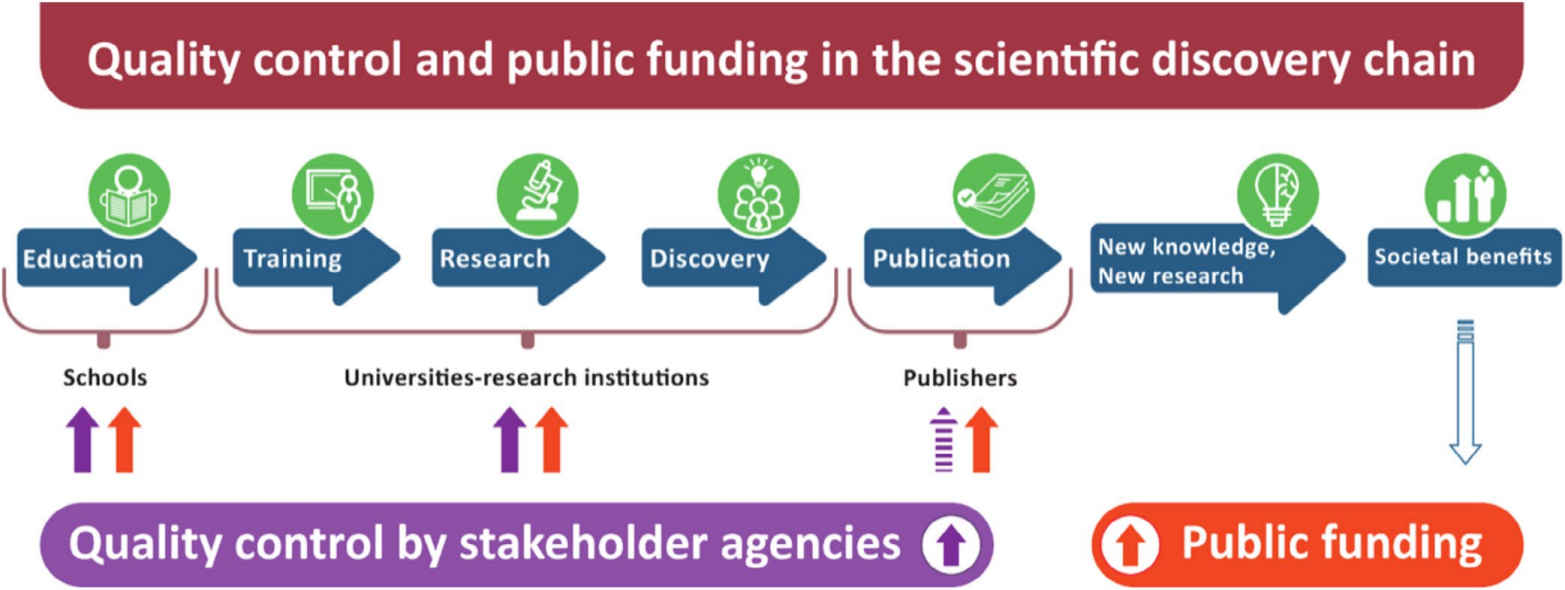
The taxpayer funds and is assured a high level of quality control of the entire chain of the research endeavour … except at the vital last stage. For simplicity, this scheme ignores private investment in education and research and development. The key elements are that public investment pays entirely or in part for not only the entire discovery chain, including the mostly not‐for‐profit public institutions housing it and their infrastructure, and the various agencies involved in managing and funding such institutions, but also for the publication of discoveries in research journals which are mostly commercial products of for‐profit enterprises. Until recently, rigorous mechanisms of quality control vital to the acquisition, dissemination and exploitation of new knowledge were imposed by experts of the research ecosystem at various levels in all stages of the scientific discovery chain. This included the key quality control mechanism for publication, namely “gating” by rigorous peer review of a submitted manuscript—the final product of the research chain. However, gating is the responsibility of journals and their editors, and how seriously journals take this responsibility is essentially optional. In the case of journals with predatory practices, the importance and quality of gating have decreased in favour of increased profit. *Counter‐intuitively and unacceptably, agencies responsible for the disbursement of taxpayer funds that pay for research paper publication exert essentially no oversight or influence on this vital element of quality control at the end of the discovery chain*. This fundamental deficit is, however, readily remedied through the process of journal accreditation proposed in this Editorial.

Any lowering of the peer review element of science quality control reduces research publication standards and inevitably erodes confidence in the research process and hence in researchers and what they produce. This in turn will result in a reduction in evidence‐based decisions being made by the public and politicians, which would be catastrophic for society and humanity (Figure 1).

## 4 Strategies of Journals Employing Predatory Practices: The Pull

While many journals employing predatory practices fail because they lack professionalism, others are successful. There are a range of reasons for their success, but the main ones are recruiting articles from reputable scientists that boost Impact Factors (IFs) and act as attractors for other submissions that are not subjected to strict quality controls, and the massive exploitation of early career scientists as guest editors to seek submissions for Special Issues from their networks. These strategies intertwine in various ways:

- *low quality‐rigour barriers of acceptance*. Like any other profession, researchers and their research are diverse, so quality and originality range from poor to outstanding. Albeit with notable exceptions, historically medium‐to‐poor research was generally published in journals of modest standing, good‐to‐very good research in journals with good reputations, and exceptional research was published in highly selective, prestigious journals. There was a clear relationship between the standing of a journal and the quality‐originality of the work it published. No longer: journals with exploitative practices selectively offer APC waivers to leading researchers to attract very good research and boost IFs, precisely in order to attract submissions of research of variable quality and the APCs they bring. This practice leads to the publication of substandard quality‐low originality research mixed with good research. This in turn confers by association the impression, particularly among less experienced researchers, that the poor research is better than it is and in turn lowers quality expectations and ultimately favours the propagation and expansion of poor science. It may also, unfortunately, confer the impression that any high‐quality papers published alongside the poorer quality papers may be less impactful/rigorous than they are.
  Non‐English native speakers face additional challenges in the acquisition of the scientific reporting skills needed to write scientific papers in English that conform to journal standards and convince reviewers. Thus, they may be tempted by the lower acceptance barriers of journals with predatory practices to publish very good work in them.
- *the appeal of rapid acceptance*. The buzz scientists enjoy is doing new science: writing papers is going over old science, and the process of publication, which may involve the need to carry out further studies to satisfy reviewers' concerns, and/or re‐writing and submitting to multiple journals, with the ‘loss’ of precious time and resources, can be highly frustrating. The offer of rapid acceptance may therefore be appealing, even though the appeal is misplaced. Even rigorous authors who are overburdened with tasks and have insufficient time and/or resources to respond to detailed requests for improvement and additional experiments demanded by journals with rigorous peer review systems may succumb to the appeal of rapid acceptance. We have emphasised above that rigorous reviewing is generally incompatible with rapid editorial handling. Rapid acceptance is therefore made possible in most cases through inadequate reviewing, reviewing by inexperienced reviewers, or improper editorial handling. Some journals attempt to achieve rapid acceptance rates through aggressive pressure on reviewers and editors—sometimes by daily bombardment with mails—to make a rapid decision.
- *Constant blanket bombardment of the research community with requests for submissions*. These are frequently requests for any submissions, usually linked to a previous publication of the targeted researcher, often not even on topics related either to the cited paper or to the title or scope of the journal.
- *Encouragement of authors lacking significant or adequate expertise in the field to submit review articles*. Journals with predatory practices aim to persuade researchers to submit almost any type of material subject to APC charges. Historically, review articles were mostly contributed by researchers with documented experience in a particular field and familiarity with its literature. In general, they were solicited by journal editorial teams targeting qualified experts expected to bring new insights to the field and to fill gaps in the literature. No longer: journals employing predatory practices have now created open spaces that enable researchers to submit review articles—recruited and unrecruited—even if their knowledge of/expertise in the field is limited/inadequate. Such reviews, while generally of little value, generate significant APCs for the journal. Moreover, because their titles and abstracts may indicate treatment of an important topic, and many scientists do not have the time to read much more than titles and abstracts, they may be cited more than they merit because they are perceived to represent the latest information, thereby contributing to the journals' IFs and other metrics. This practice is likely to be amplified with increasing use of artificial intelligence and contribute significantly to the general problem of excessive scientific publication.
- *The special case of special issues*. The idea behind a Special Issue is that it is *special*, that is, provides significant added value over and above that of a regular issue, for example by collecting high quality articles on a pioneering topic that has not been widely covered, or by bringing together articles that are ordinarily published in journals from different fields but on topics that meaningfully connect and thereby establishing a poorly appreciated connectivity. A key quality of Special Issues should be the provision of new insights. An example of such a Special Issue some of us were involved in is one in the journal *Microbial Biotechnology* on the topic of *The contribution of microbial biotechnology to sustainable development goals* (https://enviromicro‐journals.onlinelibrary.wiley.com/toc/17517915/2017/10/5). This was a pioneering issue bringing together and revealing all manner of microbial technologies and research impacting the SDGs soon after they were published. It took considerable time for other microbiology journals/societies to appreciate the importance of this and raise the flag. However, a major strategy of journals employing predatory practices to recruit submissions is to publish Special Issues non‐stop, usually involving the recruitment of young (hungry) researchers as Guest Editors and relying on them to recruit submissions from their networks, sometimes with less‐than‐optimal quality control. While this is obviously a highly successful strategy to obtain revenues (although it is beginning to face headwinds, see https://www.snf.ch/en/g2ICvujLDm9ZAU8d/news/the‐snsf‐is‐no‐longer‐funding‐open‐access‐articles‐in‐special‐issues), the Special Issues produced are rarely *special*. Criticism of the practice of organising Special Issues is increasing and justified—their image has become seriously tarnished—which is unfortunate because Special Issues can, when well done, provide new perspectives, galvanise new research directions and serve as unique informational resources.

## 5 Direct Channelling of Taxpayer Funding of Science to For‐Profit Publishers: The Issue of Cost Externalisation

There has been much discussion of the financial models of publishers of research—predatory and non‐predatory—including whether or not reviewers, the enablers of editorial decisions, should receive an honorarium for their services. Since authors of fiction and non‐fiction books, etc., receive honoraria or royalties from publishers, it may be legitimate to pose the question: should authors of papers published in research journals, or perhaps the institutions in which they work, also receive some level of reimbursement for their contributions (https://www.theguardian.com/science/2017/jun/27/profitable‐business‐scientific‐publishing‐bad‐for‐science)?

However, the purpose of this discourse is not to enter into this discussion but rather to emphasise the issue of *cost externalisation*. “Externalised costs are costs of production that someone else pays.” (https://www.imf.org/en/Publications/fandd/issues/Series/Back‐to‐Basics/Externalities; https://realitysandwich.com/sacred_economics_chapter_10/). In the case of the costs of research publication, the taxpayer pays for the researcher, the research itself and its infrastructure, including (some of) the time and effort spent in writing papers and the (mostly repeated) submission process and, when a paper is finally accepted, the APC. In return for the APC, the publisher provides the service of editorial handling and publication and archiving of accepted papers. Since most original experiments fail, with only a few succeeding to advance knowledge frontiers, and leading to publication, the publisher in fact takes “the cherries on the cake”, with no risks or losses resulting from the research failures. In fact, none of the costs of the process of creating the product publishers sell are borne by publishers (Bergstrom 2001; Trueblood et al. 2025); all are “externalised”. The unusually high profitability of science publishing is a direct result of cost externalisation. This issue is becoming increasingly relevant with publishers perpetually raising the cost of APCs while research budgets flatline or, in many countries, decrease. As a result, the *proportion* of research budgets delivered to publishers is in many cases increasing as the proportion delivered to research itself is decreasing. This trend is unjustified, of considerable concern and clearly unsustainable.

There is a need for a re‐evaluation and re‐set of the financial relationship between funders of research and publishers, in order that publishers begin to make a fair contribution to the process that yields the product they market, a return of some of the profits to the research ecosystem. How a re‐set might look—payment of authors and/or reviewers for their services to publishers, payment to the research organisations in which the work was done, payment to funders of research, etc.—is for the involved stakeholders to consider. However, given that many scientists work considerably more than the statutory working hours for which they are paid, performing research, writing and submitting their papers, and reviewing those of others (see Aczel et al. 2021, for estimates of the value of *pro bono* peer reviewing), it may not be unreasonable to envisage a reset involving in part some remuneration of authors and reviewers.

## 6 Are Mainstream Journals and Publishers Moving Towards Predatory Practices?

The financial success of journals with predatory practices appears in some cases, despite pushback by principled editors, to be inducing some mainstream journals to relax the rigour of tried and tested editorial practices. This is often initiated in publisher:editor strategy meetings with a statement from the publisher like: *Many submissions are borderline in quality‐originality, so the acceptance/rejection decision in such cases may sometimes be rather arbitrary. Accepting more borderline papers would not reduce either* the *overall quality of the journal or its IF, but would increase its size (*aka profit*) and standing in the field*. While accepting that there may be difficulty, and hence some subjectiveness, in editorial decisions on borderline papers, the quality‐originality acceptability threshold in most journals has been defined by editors and implemented by editorial board members‐reviewers. Arguing for acceptance of more borderline papers is simply a strategy to lower established quality thresholds. It is important to emphasise that this tendency is not limited to for‐profit publishers: it is also affecting journals of some not‐for‐profit organisations, including learned societies, that traditionally are significant actors in the maintenance of the science ethos and that should know better.

## 7 Predatory Journals and Science Media

Science media are an important link in the chain that is frequently overlooked in the conversation. They take the output of scientific research (the journal papers) and translate it into articles and content that are digestible and comprehensible for the wider public—those who ultimately fund a lot of the research. While some science communicators may themselves be or have been scientists, many come from an educational background of English or other languages, creative writing and journalism. And even among those who have scientific experience, it is highly unlikely they can possibly be experts on every research topic that they cover. Add in time pressures and targets, and the need to cover topics that may have wider appeal, and it is clear that there is a need for clear signposting as to what research warrants their attention. Peer review by those who do have in‐depth knowledge of that specific field, and gatekeeping by journals, are the vital quality control steps that should be preventing poor‐quality research from ever reaching the communications desk (Anderson and Ledford 2021). As long as predatory journals exist that output poor‐quality science, involving science communicators in and arming them with the tools to identify good research from bad (like accreditations to know where they should be looking for good research that warrants airtime), while choking off poor science and not giving it a voice, is also a key aspect of this issue.

## 8 Newer Publication Models

In recent years, new publishing models have been launched (e.g., https://elifesciences.org/inside‐elife/54d63486/elife‐s‐new‐model‐changing‐the‐way‐you‐share‐your‐research; https://peercommunityjournal.org/page/about/; https://open‐research‐europe.ec.europa.euhttps://f1000research.com/about; https://researchoutreach.org/outreach‐leaders/article/towards‐a‐new‐model‐of‐academic‐publishing/; Trueblood et al. 2025), some of which have commonalities with preprint servers (https://asapbio.org/preprint‐servers) in that unreviewed versions are posted with a digital object identifier (DOI), but that may also involve posting solicited reviews or commentaries (https://www.copyright.com/blog/understanding‐the‐role‐of‐preprints‐postprints‐the‐version‐of‐record/; https://scholarlykitchen.sspnet.org/2022/02/14/the‐state‐of‐the‐version‐of‐record/). Some abolish the accept/reject decision, allowing authors greater control to publish papers as their indexed version of record without necessarily addressing all reviewer concerns. While most of these new‐generation publication platforms charge APCs, some do not (https://peercommunityjournal.org/page/about/). For example, the *Open Research Europe* publication platform of the European Commission does not charge authors; however, it only accepts reports of research funded by the Commission (https://open‐research‐europe.ec.europa.eu).

While journals pioneering these approaches may have quality control measures in place and ensure comments from reviewers and author responses are made publicly available to readers, assessment of the value and rigour of such papers by interested researchers relies on them taking the time to read both the papers and the reports, which increases the already high burden of information assimilation. Moreover, searches by search engines using artificial intelligence (AI) may result in taking the paper at face value or skimming the abstract without the context of the commentary.

Importantly, it remains to be seen whether wider adoption of the newer publication models will solve key problems of research information dissemination. Indeed, a restriction of operating space of exploitive practices is not immediately evident; on the contrary, it may well be that these may flourish in such an environment.

## 9 Management Practices in the Research Ecosystem Drives Submissions to Journals With Predatory Practices: The Push

Human characters are highly diverse and cannot be readily influenced. What can be influenced is the *operating space* available for their expression; in particular, the expression of bad behaviour can be constrained by reduced operating space. In the case of those managing and editing journals employing predatory practices, there is essentially operating space without limit. In order to restrict operating space for journals to a healthy level that promotes and maintains best practice in science and its reporting, it is essential to identify and address the reasons why current operating space is essentially unrestricted. The success of journals with predatory practices is, in part, due to the fact that they seemingly respond to needs created by systemic failings in the organisation and operation of the research ecosystem that have emerged over the last few decades (Casadevall and Fang 2024). Through the identification of such failings, we can devise corrective measures that halt the downward spiral and eventually lead to the restoration of a quality control chain that ensures research rigour and excellence. These failings include:

*Publication funding*. A major problem for some researchers that drives them to journals with predatory practices is that they are mandated to publish in open access journals while lacking sufficient publication funds to do so. Many of them must pay for publication from their grants (or, in some cases, from their own pockets; e.g., see Kamerlin et al. 2021; Frank et al. 2023). In such cases, funds for publication may not be ring‐fenced or, if they are, may be deliberately inadequate in order for the grantee to have the maximum available for the research itself in situations where grants have a funding ‘ceiling’. Many research projects are in any case underfunded and overcommitted in terms of aims, effort, time and costs. Therefore, given the choice between paying between $ 2000 and $ 10,000 for publication of a paper, or being able to buy essential consumables by accepting a publication waiver from a predatory journal seeking to leverage the paper to attract APC‐paying submissions, many researchers opt for the latter (e.g., see Kamerlin et al. 2021).

The obvious solution to this dilemma is to separate research grants from publication funding and thereby remove the incentive to accept APC waivers in order to eke out research funds.

**The dilemma and discrimination caused by grants that combine costs for research and publication**

The combination of being obliged to publish in open access journals and to pay APCs from research budgets creates for affected researchers a massive and unnecessary dilemma. It also constitutes a huge discriminatory asymmetry between researchers in funding systems that separate financing of research and publication costs and funding systems that do not.

In fact, this dilemma is recognised in an explicit recommendation of Plan S Principe 4: “*Where applicable*, Open Access publication fees are covered by the Funders or research institutions, not by individual researchers; …” (https://www.coalition‐s.org/addendum‐to‐the‐coalition‐s‐guidance‐on‐the‐implementation‐of‐plan‐s/principles‐and‐implementation/). While a number of agencies indeed separate the funding of research and publications resulting from it (e.g., see https://www.jisc.ac.uk/next‐generation‐open‐access; https://wellcome.org/grant‐funding/guidance/open‐access‐guidance/open‐access‐funding), because this is an option rather than an obligation, some funders of academic research improperly choose to ignore it. In enthusiastically embracing Open Access without fulfilling this essential condition, such funding agencies fuel predatory publishing.

A number of scientists and organisations have identified and expressed concern about shortcomings of Plan S (e.g., Haug 2019; Mann 2019; Kamerlin et al. 2021; Petrak et al. 2021; https://sites.google.com/view/plansopenletter/open‐letter; https://forbetterscience.com/2018/09/11/response‐to‐plan‐s‐from‐academic‐researchers‐unethical‐too‐risky/#comment‐22170). One major aim of Plan S is a moderation of publisher charges: “Hence, driven by our duty of care for the proper functioning of the science system, we have developed Plan S whereby research funders will mandate that access to research publications that are generated through research grants that they allocate, must be fully and immediately open and *cannot be monetised in any way*.” (https://www.coalition‐s.org/why‐plan‐s/), and “cOAlition S will thereby contribute to establishing fair and reasonable prices for publishing services, including equitable waiver policies, *that reflect the publishing costs*.” (https://www.coalition‐s.org/addendum‐to‐the‐coalition‐s‐guidance‐on‐the‐implementation‐of‐plan‐s/principles‐and‐implementation/). The goals “*cannot be monetised in any way*” and “*fair and reasonable prices for publishing services*” would seem to be distant realities (https://www.theguardian.com/science/2017/jun/27/profitable‐business‐scientific‐publishing‐bad‐for‐science; Khoo 2019; Alwine et al. 2021; Alonso‐Álvarez et al. 2024). Rather, *monetising in any way* seems to be the result and publisher policies to continually raise APCs are emboldened.

A particularly disturbing aspect of Plan S is Section 7 of its *Principles and Implementation* (https://www.coalition‐s.org/addendum‐to‐the‐coalition‐s‐guidance‐on‐the‐implementation‐of‐plan‐s/principles‐and‐implementation/): *Compliance and Sanctioning* “The individual members of cOAlition S will align their grant agreements and/or contracts with Plan S and monitor compliance and sanction non‐compliance. Each funder will determine how best to monitor compliance and what sanctions to introduce. *Possible sanctions could include: withholding grant funds, discounting non‐compliant publications as part of a researcher's track record in grant applications, and/or excluding non‐compliant grant holders from future funding calls*.” These suggested sanctions could readily, immediately and irrevocably destroy researcher careers and hence seem seriously disproportionate. Importantly, there seems to be no equivalent monitoring of compliance and sanctioning of non‐compliance of the participating agencies of cOAlition S who fail to fully implement the principles and practices of Plan S. This asymmetry between imposers and imposed is unfair and unreasonable, and, if sanctions are imposed, it may constitute a potential failure of duty of care responsibility by the agencies involved. Some might also consider it to be discrimination. Any agency that adopts Plan S but fails to implement key elements, in particular the separation of funding research and publication, and the requirement to fully fund publications, should seriously consider whether it has legitimacy.

To address these important issues, there is an urgent need for an independent oversight agency that

- regularly evaluates the validity and success of Plan S strategy and aims,
- develops measures to optimise success and correct failings, in particular those resulting from the diversity of policies and practices of the different funders in the alliance,
- ensures that participating agencies of cOAlition S recognise and fulfil the responsibility of duty of care they have for those that they fund, in particular relating to alignment of what they demand and what they enable,
- monitors participating agencies of cOAlition S for compliance,
- ensures that, if sanctions are to be imposed for lack of compliance (of both funders and fundees), they are proportionate,
- prohibits funding agencies from mandating open access publication if they do not fulfil essential requirements, such as adequate funding of publication independently of research grants.

An update of Plan S was recently proposed (https://www.nature.com/articles/d41586‐023‐03342‐6): it remains to be seen whether or not it will deal with the shortcomings alluded to above and have a significant impact on predatory publication practices and the costs of publication.

*The* “publish or perish” *culture of the academic career*. Institutional management of academic career progression, the awarding of research grants, invitation of speakers to present work at conferences, appointment to influential committees and, to a considerable extent, acceptance of papers by prestigious journals are, despite pushbacks by organisations like the Declaration on Research Assessment (DORA; https://sfdora.org; https://sfdora.org/read/), the “Leiden Manifesto” (Hicks et al. 2015; https://www.universityworldnews.com/post.php?story=202108271321513) and a few universities (https://www.universityworldnews.com/post.php?story=20220511170923665), largely driven by publication metrics, especially publication quantity and IFs of the journals in which research is published: the “counting mindset” (Trueblood et al. 2025; Dougherty and Horne 2022). Critical assessment of the importance, originality and long term impact of work is largely ignored because (a) it cannot be done by managers/administrators who like to be in charge of career development and research policy, (b) managers/administrators may not like the answers provided by experts, because they may not understand the assessment process, and (c) comparison with other groups and institutions (rankings) may be difficult. Thus, quantity not quality, and publication metrics, dominate management of the research ecosystem (Fang and Casadevall 2012).

This begins with the Ph.D., the awarding of which in many countries includes a requirement for first author peer‐reviewed publications. In some cases, at least three such publications are needed for the degree to be awarded. The spirit of this practice, which is intended to bring greater objectivity to research assessment and counter ‘local’ personal preferences and interdependencies, is laudable. Nevertheless, it already introduces the notion of quantity instead of quality, of three papers being acceptable whereas one (which may be outstanding) is not, and also risks inadvertently fuelling scientific misconduct. In some cases, it is a practical induction at the outset of a research career into *salami‐tactic* or *least publishable unit* publication practice—the tendency to split up a research work into small elements, rather than presenting the story as a whole. In some institutions, it is recommended that the thesis introduction is also considered for publication as a review/minireview. Given that a central tenet of publication is that new information/insights should be provided (otherwise it has little purpose, and may be considered to be publication for its own sake), and that a thesis introduction is almost always a summary of relevant literature, with few new insights for experts, this practice makes little sense and should be discouraged. (This is not meant as a criticism of Ph.D. students: a Ph.D. project is all about training to become an independent researcher—a 3–4 year training period to gain the experience needed to interpret the results and conclusions of others—so profound new insights are the exception rather than the rule.) As a result of this practice, the literature is unnecessarily littered with an enormous body of reviews that say little of value but represent a significant amount of taxpayer funds.

**The struggle of time constraints and mandatory papers for Ph.D. students**

Since many Ph.D. projects are time‐ and/or funding‐limited, the requirement for accepted peer‐reviewed papers as a condition of the awarding of the degree can create intense pressure and anxiety in students who need them to graduate and apply for a follow‐on postdoctoral position. A looming loss of livelihood combined with an inability to seek new employment can readily translate into submission of incomplete papers to journals with predatory practices. *Thus, counter‐intuitively, in the current circumstances*, *a mechanism intended to raise standards may in effect result in their lowering*.

However, this is merely the tip of the iceberg, because it is just the beginning. Advancement in the academic career obliges adoption of the *publish or perish* mantra increasingly imposed by the academic system, university/research institutes and governments (Trueblood et al. 2025). This mantra, coupled with the easy publishing offered by journals with predatory practices, is increasingly leading to the publication of incomplete work, results that have not been adequately demonstrated to be reproducible (e.g., Casadevall and Fang 2010; Munafò et al. 2017), unoriginal reviews that fail to meaningfully advance insights or understanding and, in some cases, papers reporting fraudulent work that if/when it is detected, must be subsequently retracted (Martin 1992; Fang et al. 2012; Gross 2016; Van Noorden 2023; https://www.bangkokpost.com/thailand/general/2831668/six‐academics‐sacked‐for‐research‐paper‐fraud). It has provided fertile ground for paper mills (Brundy and Thornton 2024; Parker et al. 2024) and review mills (Oviedo‐García 2024), which in turn have massively increased the flux of publications.

*Development of a laser‐sharp focus on high‐impact journals, proliferation of sister journals and cascading*. Because of the career development importance of the IF of the journal where a paper is published, authors often submit (“just in case”) to a journal of relevance with the highest IF, often with a considerable disparity between journal standing and the importance of the work reported. In many cases, the to‐be‐expected desk rejection is not immediate but significantly delayed. A desk rejection implies that the standard of the work is clearly apparent to the editor as being lower than that required by the journal. It should therefore be made promptly, both because it can and because it avoids unnecessary wastage of precious time and allows the authors to move on. Extended delays are unconscionable, often unethical and cause significant author frustration.

Crucially, publishers are increasingly creating journal families associated with a high IF parent journal and cascading submissions down the IF ladder within the family. When the rejection eventually comes, it often comes with an offer to cascade to another journal in the family. This is often simply to avoid releasing the paper to a competitor/to retain the possibility of collecting the APC. Because this offer gives hope of associated prestige, authors often accept. The process may then be repeated, sometimes through multiple journals of the family, resulting in an extremely long waste of time. Ultimately, authors may become so frustrated that they give up and submit to a predatory journal with low acceptance standards. This hold‐reject‐cascade strategy is unethical, to be discouraged and another factor favouring journals with predatory practices.

*Distortion of the vital interface between researchers and research managers*. Although in some cases research managers in funding agencies, research institutions, science policy, etc., may also be scientists who understand the issues underpinning good science, in other cases managers have no scientific background. Over time, even scientist managers become less familiar with progress and new scientific challenges, opportunities and priorities. Thus, managerial practices and decisions in research are dependent upon input of information and suggestions from scientists: the vital scientist:manager consultation interface. However, many managers feel uncomfortable and inadequate discussing issues about which they have insufficient knowledge, and hence a dependency on others, so often gravitate to discussion partners who can make them feel at ease. This leads in some cases to interfaces between managers and scientists who can *talk the talk*, but who do not, and in some cases are not able to, *walk the walk*. In order to further their own career, some of these scientists talk up second‐rate, unoriginal and uncreative ‘science’ they do themselves. Some of them also avail themselves of the easy publication route of journals with predatory practices in order to document at opportune moments their own contributions to the research they advocate. This system of “self‐service” leads to a channelling of significant resources into pedestrian science and a starvation of resources for original ideas that lead to major advances. The funding of pedestrian science leads to its citation via group‐thinkers and the unwarranted career rewards this brings. This in turn creates a group of mentors for the next generation of researchers and hence perpetuation of poor science and the erosion of good science.

**The vital interface between researchers and research managers**

Science managers have the responsibility to their paymasters and the wider public to promote excellence in science and obtain best value for money. Since most of them lack the knowledge vital to the decisions they take in this endeavour, they have the responsibility to seek and implement the very best advice available. Academies representing leading scientists should be a default source of guidance in these vital interfaces.

*Countries with less well‐developed research ecosystems*. A significant portion—perhaps 80%—of scientists worldwide lack the resources and technological infrastructure available to the approximately 20% in scientifically advanced regions. These disparities impact access to reputable journals and project funding. Additionally, scientific education policies and academic promotion criteria in some countries may fail to foster a rigorous research culture. Instead, legislators—who may lack an understanding of the importance of scientific integrity—may push populist policies that align with flawed ranking systems and publication in journals with predatory practices. For instance, universities and research institutions may sign agreements with publishers of journals with predatory practices merely to meet open‐access requirements for research funding. This creates a system where scientists—both young and established—feel pressured to publish in journals with rapid acceptance/publication schedules and low peer review standards, particularly because prestigious journals may take months to review papers, sometimes rejecting them because of trivial details. As a result, important discoveries occasionally appear in predatory journals—not because researchers prefer them, but because of systemic constraints and sometimes a coercive career framework.

It is essential that reviewers and editors recognise that science in such countries is often not supported with the latest technologies or funding sufficient to go the extra mile that may typically be required by reviewers, but can otherwise be rigorous and insightful. Scientific proficiency within a country requires time to mature in order to create a proper structure of self‐evaluation and financing. During this developmental period, conditions can be such that researchers have few, if any, opportunities to publish in highly reputable journals. It is crucial to recognise that, in some cases, journals with predatory practices offer authors a vision of an opportunity that is truly needed. In any case, the pressure to publish in open access journals without an effective mechanism to adequately cover the associated APCs (see some of the cases described in Shen and Björk 2015, and references therein) widens the existing inequity and increases the discrimination between scientists in well‐funded and less well‐funded settings (see Trueblood et al. 2025 for a synthesis of relevant issues).

There are also examples of editors of some non‐predatory journals chasing higher IFs being instructed by publishers or learned societies to desk reject submissions of acceptable quality standards that may be deemed unlikely to be well cited. This form of discrimination can also drive authors to submit papers to journals with low standards of acceptance.

*Employer distortions of merit‐based career development and researcher income: publication practices influenced by inducements and pressure*. Some universities pressure researchers to increase the number of their publications by ranking them in applications for academic posts, promotions and/or in some cases for payments additional to normal salaries (“performance payments”, see, e.g., Hedding 2019; https://scholarlykitchen.sspnet.org/2011/04/07/paying‐for‐impact‐does‐the‐chinese‐model‐make‐sense/), according to the number of papers published. In some countries, this has led to the emergence of “paper mills”, organisations that produce fake manuscripts on an industrial scale and provide authorship for a fee (Brundy and Thornton 2024; Parker et al. 2024).

Some universities operate a “publication scores” or “points” system based on, for example, the IF and/or the quartile range. The score of each paper may then be divided according to the number of co‐authors and the order of applicant within the author list. This practice encourages academic staff in such countries to publish more papers with fewer co‐authors, in order to achieve higher scores from publications. Quite apart from other considerations, this obviously constitutes an undesirable distortion of the collaborative nature of science. Since the quality of the research or the publication may have secondary importance in such systems, the practice encourages unambitious research by small groups and its publication in lower quality journals (see also the issue of publication inflation below). Point systems may operate at both local and national levels, for example, in national applications for professorial appointments.

*Political distortion of merit‐based research funding and career development*. In some countries, research funding may be strongly influenced by the political affiliations of applicants for grants, resulting in extreme cases of over‐funding of poor‐quality research and under‐funding of excellent research. A knock‐on effect of this is the inability of good researchers to complete some of their work in the manner prescribed by good scientific practice, to publish it in good journals demanding rigour and completeness, and thus having limited options for publication of their work. This results in a significant wastage of funding.

Perhaps even more importantly, employer and political distortions of merit‐based advancement of researchers drive some of the best to seek employment in more equitable settings. Since economic development is promoted by a strong research base, the loss of creative, original research talent through wastage and brain drain, and the underfunding of some of the best talent/overfunding of some of the mediocre talent that remains, can result in serious economic underperformance to the detriment of an entire country.

## 10 The Value of Publication and Its Devaluation by Inflation and Deteriorating Quality

Publication of research advances is the means by which knowledge is transmitted among scientists and has immense importance to science and the wider world. It represents the fruits of enormous investments by the taxpaying public. For publications to have legitimacy, they must report meaningfully new and rigorous findings that endow them with value that justifies the investment underlying their creation. Rigorous findings are those that have been shown by the authors to be reproducible; this takes time. Prolific production of papers often reflects inadequate reproduction, reproducibility and hence rigour. Papers lacking rigour, reporting unoriginal work, incomplete work or fraudulent results (https://www.bangkokpost.com/thailand/general/2831668/six‐academics‐sacked‐for‐research‐paper‐fraud) and/or produced by “paper mills” (Brundy and Thornton 2024; Parker et al. 2024), lower the value of publication and its legitimacy (MacLeod et al. 2014).

Of deep concern is the current veritable explosion of publications of low value (Conroy 2024; Ioannidis et al. 2024; https://www.universityworldnews.com/post.php?story=20180905095203579). This is driven, on the one hand, by predatory publication practices and, on the other, by trivial parameterisation by non‐scientist managers who have little idea of the scientific process and how originality and scientific productivity likely to lead to significant advances can be assessed, which has created the “publish or perish” culture that is prevalent in many research establishments.

*Publication inflation compromises the ability of scientists to be up to date*. For researchers to conceive research that moves knowledge boundaries forward, and to avoid undertaking research that has already been done elsewhere or whose utility is doubtful, they must know where current boundaries are, that is, be up to date. Similarly, to be effective, fair and constructive reviewers, they must also be familiar with current knowledge. However, the current rate of publications is totally incompatible with the time available to scientists to read and critically assess new papers, and the number that need to be read. One study reports that the total number of articles published in journals covered by indexing services is increasing exponentially (Hanson et al. 2024). Another suggests that between 2010 and 2015, the number of articles published in journals with predatory practices increased from 53,000 to 420,000 (Shen and Björk 2015). This rising tide of publications is unsustainable in the context of the essentiality of awareness of the latest literature needed for original ideas, rigorous peer reviewing, keeping up with relevant literature and research‐publication funding that is flat or only marginally increasing.

*The key issues of time and mental framework for creativity*. New original ideas that drive scientific advances and excellence are not items conveniently programmable into busy schedules: their appearance is unpredictable. That said, light‐bulb moments are usually the result of multiple episodes of focused consideration of an issue/problem at times when other activities, pressures and mental demands take a back seat, and receptivity, the open state of mind that allows the new idea to crystallise out. In general, creativity is excluded by other activities; the mind and its mental processes need operating space. It is not for nothing that individuals often say that an idea came to them while showering, or that groups synthesise a new concept over drinks in a bar, when they are collectively focused on a problem with no other mental intrusions (intellectual “ping‐pong” among a group of experts). Publication takes an enormous amount of work and intellectual effort. This, coupled with all the other activities and responsibilities of the academic, conspires to constrain the time, opportunity and mental framework essential to the process of creation. The proliferation of publications is a major barrier both to being up‐to‐date and to the emergence of new ideas and advances in excellence. Science managers who develop and administer frameworks for creative research, but who are not themselves expected to be creative, need to understand this fundamental difference and its relevance to their own success.

*The problem of finding willing, qualified reviewers*. High‐quality peer review requires considerable time and effort by researchers. The rapidly expanding rate of journal submissions is engendering a rapidly expanding need for peer review activity at a time of flatlining of academic research budgets and personnel in many countries. Budgetary constraints generally trigger increased workloads of already over‐burdened and over‐committed scientists, with the result that they have less, rather than more, time for reviewing. A frequent complaint of authors is a frustratingly long editorial handling time, which, although sometimes warranted, usually results from a difficulty in finding willing, qualified reviewers. Resolution of this conundrum requires a multipronged approach involving a reduction in the number of publications through imposition of higher standards, a reduction in unreasonable and unsustainable workloads of academic researchers to allow a return to earlier levels of reviewing, and employer recognition of the value of peer reviewing and its incentivisation by appropriate recognition in career development procedures.

**Consequences of publication inflation**

- reducing time and mental space for creative thinking needed for new discoveries,
- dilution of good work and blurring of distinction between original, challenging research and pedestrian, easy quasi‐repetitive research (“data points”),
- normalisation of *salami tactics* implies acceptance, which degrades the notion of value,
- increasing limitation of what proportion of relevant literature can be read and resulting increasing focus on work only within one's own field of activity,
- decreasing ability to connect with important relevant findings outside of a narrow field of activity (i.e., an increase in “silo” thinking and diminution of systems thinking), findings most likely to trigger creative new ideas that can lead to the most original research and innovations,
- reduction in competent research at disciplinary boundaries, which is often highly productive,
- diminishing knowledge needed for critical and fair reviewing,
- reduction in the already limited expertise needed for reviewing interdisciplinary work,
- increasing numbers of publications requiring increasing numbers of reviews at a time when reviewer availability is flatlining or reducing creates space for “review mills” and predatory practices that include insufficient or unserious reviewing,
- increasing tax revenues unnecessarily flowing to publishers (e.g., Moher et al. 2017; Haug 2019; Khoo 2019).

All of this contributes to the decreasing value of publication. There is an urgent need for all actors in the research ecosystem to push back against any lowering of publication value and to enthusiastically promote the mantra “publish less and publish better”.

## 11 The Uncomfortable Issue of Our Own Culpability

It is not our purpose to point fingers but rather to explore and encourage implementation of remediation options by people and agencies that have both the responsibility and means to take actions that can reorientate science communication practices onto a healthier trajectory. However, because of other preoccupations, there is considerable inertia among the actors who need to act. It may therefore be instructive to consider the issue of culpability because recognition and acceptance of culpability may help galvanise action.

Firstly, we scientists are culpable because some of us publish in, review for or are editors of exploitative journals and thereby nurture and legitimise them. There are diverse reasons for this (Beall 2012; Frandsen 2019), but the involvement of senior scientists in editing and authoring papers may in some cases be driven by a perception of elevated standing as an editor, guest editor of a Special Issue or author of a review (e.g., Franck 1999). The related issue of self‐esteem can also be a factor because predatory journals provide opportunities to researchers who have difficulty profiling themselves, especially those from countries with less‐developed research cultures/infrastructures/histories.

These practices signal to mentees and younger scientists that predatory journals are acceptable, sometimes even preferred vehicles for publication. Others who refuse to publish in such journals nevertheless do not call out, or call out sufficiently pointedly, those who do. And many of us do not instruct the young scientists we are mentoring with forceful and unequivocal statements. So: scientists are culpable and we need to take stock and actively correct the situation.

Secondly, the many enthusiastic proponents of open access publishing, and in particular Plan S, which insists that funding agencies mandate recipients of funds to publish in open access journals, without due consideration of the possibility‐likelihood that this could create a marvellous opportunity for the development of exploitive business practices are culpable (Beall 2012). (*To avoid any misunderstanding: the authors of this editorial are fully committed to the principle of open access publication. But not with the degree of freedom for abuse that characterises current systems*.).

Thirdly, some funding agencies—both public and philanthropic—which mandate grant recipients to publish work they support in open access journals, without ensuring that a quality control system for such journals is in place (Beall 2012), directly channel funds to publishers with predatory practices. Thus, some funding agencies are culpable.

Fourthly, relevant government departments (science, health, education, enterprise, business, etc.) that set or influence science policy and decide upon research budgets have a direct responsibility to constituents and taxpayers to ensure that the highest scientific standards are maintained and that tax revenues are not wasted. Thus far, most have not intervened or effectively intervened to counter publication in predatory journals, so they are also culpable.

Fifthly, universities and publicly‐funded research institutes—the homes of academic research—have both a responsibility to impose, monitor and ensure the highest benchmarks for scientific rigour and quality of their research outputs, and a duty of care to their researchers and the young scientists they mentor to ensure they are properly guided and supported. But, even more importantly, centres of academic research and learning need to examine the culture they have created for researcher/academic career development, especially reliance on publication metrics to measure ability and productivity, which in many instances is faulted but nevertheless a driver for the spirit of *publish or perish*, which itself is a driver for the use of exploitative journals as vehicles for publication.

Sixthly, indexing services and internet search engines do not always filter out unserious journals (Beall and DuBois 2016; Severin and Low 2019), so also contribute in some way to the problem.

In the context of the above, it is important to emphasise that culpability is not black and white: it is quantitative. Anyone can make a mistake; the key question is: once the mistake is recognised (e.g., Beall 2012), is it acknowledged and a serious attempt made to correct it in a timely fashion? The longer remediative action is delayed after recognition of culpability, the greater the degree of culpability. It is not for nothing that investigations of scandals habitually include the question: *when did you/they first learn about it?* All of the actors cited above, and any others involved, need to recognise and consider their culpability and take prompt appropriate remediative action.

## 12 A Solution to the Pull: Establishing and Enforcing a Code of Conduct That Restricts the Operating Space of Journals Employing Predatory Practices

Thus far, solutions proposed to the problem of journals with predatory practices have focused on recognising and identifying them and exhorting scientists to avoid them (e.g., Amsen 2024; Beall 2012; Beall and DuBois 2016; Trueblood et al. 2025). However, while these aspects are important and necessary, they have so far failed for a variety of reasons, some of which are discussed in this paper. In essence, however, they constitute response mode approaches.

The solution we propose is the development and imposition of enforceable rules of acceptable journal and editorial practices, that is, actions that embody the principle of *prevention, rather than* the *cure* of predatory practices, and that change the situation from one of *playing catch‐up* to one of *being ahead of the game*.

**An actionable solution to the problem of predatory practices: Restriction of operating space**

This solution envisages five main action elements:

1. *Creation of a Journal Code of Conduct*, which specifies best practice for editors and journals and proscribes exploitive practices.
2. *Accreditation of journals*, based upon the submission of evidence of implementation and practice of the Code of Conduct. This places the onus of proof of lack of predatory behaviour squarely on the journals.
3. *Funding only of APCs charged by accredited journals*, which eliminates the business model of journals failing to adopt the code of conduct.
4. *Obligatory separation of research grants and APC payments by ALL institutions mandating OA, and adequate independent funding of APCs of research groups supported*. This will remove the conundrum challenging many groups of choosing between using research funds for research (publishing in a predatory journal) or for APCs in a non‐predatory journal. **Until this becomes practice, mandating such groups to publish in OA journals must be paused**.
5. *Institution of a programme to mentor early career scientists and instil the science ethos*, and to support them in ways that will satisfy their legitimate desires to gain key and citable experiences more rapidly.

The key entities able to restrict much of the operating space of journals with predatory practices are funders that finance the APCs (the nourishment of predatory journals) and indexing services that provide the journal rankings (the publication Bibles and driver of journal choice that many authors follow so diligently). The policies and actions of funders and indexing services are thus pivotal to countering the degradation of the science ethos. However, funders and indexing services are themselves diverse entities, so they need to enter into an alliance with one another and other actors to forge a common policy to reinstate best editorial practice, maintain publishing standards and disincentivise exploitative practices.

**A Journal Code of Conduct**

The purpose of a *Journal Code of Conduct* would seek to articulate certain principles that uphold the quality, rigour and integrity of scientific publications, to discourage predatory publishing practices and the publication of mediocre, faulted, unethical or manipulated works that constitute a wastage of scientific effort and public funding, and key elements of editorial best practice. We do not wish to be prescriptive, so the following list is simply some suggestions for consideration.

1. Editors must obtain expert competent reviews on submissions and make best practice decisions based upon reviews received. *APC revenue must not be a consideration*.
2. Reviewers/editorial board members must be selected on the basis of their recognised competence (e.g., see Sorokowski et al. 2017), *not on their potential and that of their network to contribute to APC revenue*.
3. Papers should only be accepted after authors have convincingly responded to all reasonable reviewer criticisms and fulfilled all reasonable reviewer requests for improvement, or provided convincing reasons why they should not.
4. The use of “Special Issues” should serve the purpose of added value, which should be evident both a priori and a posteriori.
5. Editorial decisions should be based on transparent criteria of quality and originality, *not on “citability.”*
6. The practice of “coercive citation” (Wilhite and Fong 2012) should not be permitted.
7. Cascading within journal families should only be done if the editor knows that there is a high probability of acceptance, that is, *good matching of journal requirements and the paper under consideration*.

## 13 Accreditation: The Gating of Publicly‐Funded APCs for Journals

A process of accreditation of journals should be established. A key requirement of accreditation would be unambiguous evidence of adoption and adherence to the Journal Code of Conduct. Journals may freely choose to seek accreditation or not, but access to publicly funded APCs would require accreditation. Evidence of implementation would involve appropriate standardised documentation and be based upon transparency and accountability. Journals and publishers with approved credentials would be required to maintain for a period of five years *all records* of editorial handling of all submissions (including the choice of reviewers, the reviews, editorial decisions, author responses and final decisions), details of all appointments of editors and reviewers, details of who received waivers or partial waivers and the reasons for this, etc. and make these records available upon demand by official auditors on a confidential basis. These records would enable random auditing of the practices of journals for the purpose of verification of non‐predatory practices. *It would also bring publishers into the same accountability framework in which researchers (justifiably) function* (e.g., see https://www.csic.es/sites/default/files/2023‐01/cbpc_csic2021.pdf).

All journals seeking publicly funded APCs would be required to obtain accreditation after a period of grace of perhaps 1–2 years. Because of the large number of journals that would need accreditation, the process would play out over a number of years with agency/agencies prioritising journals known to, or suspected of, predatory behaviour. A distribution of the accreditation workload between different agencies would seem the most efficient and cost‐effective, but would require harmonisation of standards and practices between agencies. Once a journal has received accreditation, it would be valid for a period of time, perhaps five years, before it would need to be renewed. Any convincing report of predatory behaviour, or for example, of multiple editor resignations (e.g., see https://retractionwatch.com/the‐retraction‐watch‐mass‐resignations‐list/), would automatically trigger investigation of the journal and, if confirmed, immediate removal of accreditation.

## 14 Making It Happen: Creation of Accreditation Agencies–Installation of the APC Gate

High‐level discussions between all stakeholders to determine how accreditation agencies should be set up, how they should operate, how they will be financed, how their work will be overseen and how their results will be used, are urgently needed. These discussions should lead promptly to the creation of accreditation agencies and the initiation of their work. Funding agencies and indexing services would need to play major roles in these discussions because they are the agencies that will use the outputs to guide their decisions. Indexing services already carry out various forms of accreditation of new journals for acceptance into their indexing systems, so for them accreditation activities would simply be an extension of existing scrutiny.

## 15 A Solution to the Push: Policy Changes at All Levels to Improve the Management and Culture of the Research Ecosystem

Predatory publication practices are in part a consequence of the wider problem of how science and academia are managed by higher organisations, the resulting culture of the research ecosystem and degradation of the science ethos. Therefore, substantive change in science management is essential to reorienting research to a more healthy and sustainable trajectory.

## 16 Creation of a Framework for a Cultural Change to Publish Less and Publish Better

At the heart of the failings of research management is the issue of how researcher career development is orchestrated, what is considered to represent success and how it is assessed, etc. (see, e.g., Casadevall and Fang 2012, 2024; Macfarlane 2023). We have outlined above, as have others, how some of the policies and practices of research institutions and funders of research promote, in some cases incentivise, or at least fail to discourage, publication in journals with predatory practices and the lowering of scientific standards this engenders. Reasons include the need to create publications on demand to meet grant funding terms, attract new funding or meet employer contractual obligations that are in some cases very target driven, ignoring the fact that not all experiments in original research go as expected and will not always generate data that make good publishable bodies of work. Academic researchers are multi‐taskers with responsibilities that may include heavy teaching loads, mentoring, administrative work, committee work, reviewing–editing and so forth. When they are pulled in too many directions, the attraction of a journal that makes little problem for acceptance of a needed publication, especially if it is not super original, creates a serious dilemma. *This poor alignment of employer expectations, researcher capacities and the science ethos is unsustainable and needs to change.*

Employers, science managers, funders and science politicians all need to recognise the vital importance of the science ethos for them and their goals, for scientists and for society, and engage in the development of fairer, more objective and differentiated criteria for merit‐based career development that prioritise originality, quality and rigour over publication metrics (see also https://sfdora.org/read/; https://sfdora.org; https://www.universityworldnews.com/post.php?story=202108271321513; https://recognitionrewards.nl/wp‐content/uploads/2020/12/position‐paper‐room‐for‐everyones‐talent.pdf; https://www.apsc.gov.au/working‐aps/information‐aps‐employment/guidance‐and‐information‐recruitment/aps‐recruitment‐guide/factsheet‐merit‐based‐decisions‐selection‐panels; Trueblood et al. 2025; https://hbr.org/2025/02/the‐false‐dichotomy‐of‐merit‐and‐inclusion). Policies and practices that encourage quantity and mediocrity must be replaced by others that are science ethos‐centric and that value and promote quality and excellence.

**Sustainability of the scientific enterprise is vitally dependent on a culture of excellence**

It is excellence in science that drives scientific advances and societal benefits (the unwritten *Society:Academia Contract* and circular economy of the scientific enterprise—Timmis and Hallsworth 2024). However, science is seriously expensive and requires long‐term financial commitments. Sustainability of the scientific enterprise is thus directly linked to and absolutely dependent upon maintenance of the culture and standards of excellence. The present trend of standard degradation will, if not promptly corrected, inevitably lead to reducing public confidence (Cologna et al. 2025) and, as a consequence, reducing financial support, especially in times of economic challenge and cultures extensively subjected to mis‐ and disinformation, such as is the case in many countries in 2025. Falls in science funding will lead to employment reductions throughout the research ecosystem and render the work of those remaining even more challenging, with a significant slow‐down of progress and resulting societal benefits. *It is vital to recognise now that this will largely be an unnecessarily self‐inflicted disaster caused by dereliction of duty by leaders in the research ecosystem. It must also be emphasised that leaders have a moral and often a statutory duty of care for their employees which, in research, involves creating a healthy workplace culture that responds to the needs of the pursuit of excellence*.

It is unlikely that essential changes will occur without outside intervention, or at least any time soon, because research institutions tend to be large, have entrenched policies and practices, established networks of influence that may not always be merit‐based and oriented, sometimes also networks of patronage, and thus suffer from inertia (see also Martin 1992).

An effective mechanism that quickly galvanises necessary change must be developed and implemented from above. Moreover, left to themselves, different institutions might develop disparate policies and practices. However, the principles of rigour and best practice in science are universal. Inventing different solutions for different countries and institutions makes little sense; a common set of guidelines is both possible and preferable, also given that scientists are a highly mobile workforce among different countries having research institutions.

## 17 The Need for Creation of a Top‐Level Independent Body to Define and Monitor Implementation of Best Practice in Academic Research: An International Alliance for Scientific Rigour and Excellence

We therefore recommend the formation of an alliance of actors whose mission is the achievement and maintenance of the highest standards in research—it could have any name but for convenience we suggest the tentative name of Alliance for Scientific Rigour and Excellence, ASRE—composed of representatives of scientific academies, organisations for research integrity (e.g., https://ori.hhs.gov; https://allea.org; https://www.enrio.eu; https://ukrio.org/about‐ukrio/), national science foundations, institutions that carry out research, like research centres and universities, researchers themselves and other stakeholders.

The task of the ASRE would include the establishment of guidelines for raising and maintaining scientific standards and the preservation of the science ethos. These guidelines would include *inter alia* recommendations on research management, conduct and ethics, publishing, reviewing and editorial activities, mentoring, support of early career scientists, employer guidelines for career development, assessment and expectations of staff, and input into accreditation agencies. A desired output of the ASRE would be the elaboration of an International Convention on the Protection of the Science Ethos, which would embody a Code of Conduct essential for the fulfilment of the Convention.

Change in the research ecosystem culture, guided by the International Convention of Protection of the Science Ethos, must be accelerated from above, through pro‐active changes in international, national and regional science policy and the funding consequences of these changes. Higher authorities must mandate the use of independent respected science experts to advise and assess productivity and value, also to avoid politicisation of appointments and funding. Specifically, government departments with responsibility for research and education policy and funding must accept the crucial importance of the science ethos, proscribe all policies and practices that lower research standards and quality, and make funding to academic research organisations they support conditional upon convincingly demonstrated acceptance and implementation of the principles of scientific best practice. They must also ensure that funding agencies they support translate such policies into action. These policy changes, if effectively implemented, could engender rapid, wide‐ranging improvements, disincentivise support of journals with predatory practices, promote career advancement assessments and decisions that are truly merit‐based and create a sustainable research ecosystem that is sharply focused on excellence and that provides best value for the taxpayer investment.

## 18 The Importance of Mentoring and Support of Early Career Scientists

The current prevalence of publication in journals with predatory practices, especially by respected, renowned scientists, lends apparent respectability and legitimacy to predatory practices and their financially motivated lowering of research standards, and seriously damages the science ethos. But, more importantly, the normalisation of publication in such journals is creating new generations of scientists that, unlike previous generations, will be unaware of the problem, accept it as the standard and will not push back. There is thus an urgent need for senior scientists to educate and mentor younger scientists in best practice, rigour, research integrity and ethics, and writing scientific papers. Young people need to become familiar with the characteristics of predatory practices and their consequences for the science ethos, not only of journals but also of conferences, and be aware of becoming involved with them as authors, guest editors or reviewers, in the case of journals, and participants, in the case of predatory conferences (Bowman 2014; Mercier et al. 2018; Das 2021; https://archive.nytimes.com/www.nytimes.com/2013/04/08/health/for‐scientists‐an‐exploding‐world‐of‐pseudo‐academia.html).

Moreover, because adequate knowledge of predatory practices and their individual/scientific harm is not universal, even among senior scientists, research institutions must also implement education for all employees about predatory publishers and motivate and financially support their employees to publish in reputable journals.

Many young researchers are desperate to acquire new skills and in need of recognition and advancement, so are susceptible to offers of guest editorships of special issues (as are, indeed, some senior scientists). These scientists in turn often reach out to mentors and other established researchers. These senior researchers, who understandably want to support their young colleagues, can thereby become co‐editors/co‐authors, thereby lending their credibility to journals employing predatory practices. It should also be acknowledged that some researchers are also deeply insecure within the publish‐and‐perish system and have deep imposter syndrome, so they are not just looking for opportunities but also any validation they can find that they have a place in academia.

The legitimate ambition of younger scientists to acquire editorial responsibilities as an integral element of their personal development and career advancement needs greater support, guidance and fostering by more senior researchers. In the process, the latter will benefit from a shared workload. A number of learned societies and journal editorial teams have integrated young people into their publishing activities, and this will certainly gain traction with time (see Casadevall et al. 2024). What is now needed is much wider engagement and mentoring of younger scientists as reviewers, editors and guest editors of respected journals, both to satisfy their justified ambitions and to channel them away from exploitative journals.

## 19 The Key Roles of Learned Societies, Their Members, Umbrella Organisations and Publications in Eradicating Predatory Behaviour, Establishing Codes of Conduct and Maintaining the Science Ethos

The primary means of associating with other scientists within a discipline in order to network, form collaborations, exchange new information, develop and influence relevant policy, help younger members and generally advance the discipline is to form learned societies. Some of these activities are mediated by society journals and at society conferences. Scientific journals were born within scientific societies, and the scientists themselves had control of the journals, defining quality standards, scope, review processes and so forth. Now, however, journals are mostly created by publishers who control the key aspects of quality standards and editorial practices. Importantly, the development of the Open Access model with APCs, combined with the elevated importance of IF, has resulted in the migration of many submissions away from society journals to journals of for‐profit publishers, some of which employ predatory practices. The value of journals of learned societies should not be underestimated (e.g., Haug 2019; Kamerlin et al. 2021).

Learned societies are usually national but, in larger countries, they may also have regional branches. National societies often also associate with other national societies representing the same discipline in umbrella organisations, such as the Federation of European Microbiological Societies (FEMS) and the International Union of Microbiological Societies (IUMS). Importantly, a learned society may typically have hundreds of members, whereas umbrella organisations will have many thousands. Many of these many thousands are mentors of younger scientists and thus play a significant role in the development of scientific standards and ethos. This represents a huge potential for influence and change, not least because some of these members are well connected to/networked with other influencers and decision makers, like leaders in politics, funding, business and education. Learned societies and their umbrella organisations thus have the ability to inform their members of the danger of predatory practices, to convince policymakers and employers to improve the culture of the research ecosystem, to provide input and guidance to organisations concerned with research integrity, and to provide attractive platforms for publication of research papers (Trueblood et al. 2025). Learned societies need to realise this potential and develop the motivation to exercise it.

Predatory journals are sometimes perceived as the only possible route to publication of work that is solid, but not “exciting”, including that reporting confirmatory and negative results, and particularly from countries in which investment in science is low. Society journals could help counter this by being pro‐active in publishing such work.

The combination of these actions could have a significant impact on the research publication landscape and ultimately help eradicate predatory practices that are a fundamental danger to good science.

**The potential of learned societies to reverse predatory practices**

Learned societies, as organisations formed to advance disciplines, have a preordained responsibility to be proactive to maintain discipline ethos and standards. If they so decide, they are able to galvanise crucial society‐level actions, such as collective public statements, providing the opportunity to individual members to sign a declaration of support, lobbying their funding agencies–science policy agencies–academic/research establishments, and so forth. Importantly, they can counter predatory publishing practices in various ways, such as making clear to their members the danger of supporting exploitive journals and the benefits of publishing in society journals (Trueblood et al. 2025). For this, they themselves must demonstrate strict adherence to publishing best practice and provide attractive conditions of publication.

## 20 Concluding Remarks

**The aim of this paper: Problem solving not blaming**

It is not the purpose of this Editorial to point a finger of blame at actors involved in or facilitating predatory publication practices. It is rather to seek a solution to an existential threat to the science ethos and rigorous research. The problem is one of essentially unlimited operating space for the development of predatory practices. Consideration of how this space may be operationally constrained leads us to propose a route back to a developmental trajectory and operating space that will ensure that the highest levels of scientific rigour are upheld and advanced: in effect a *gate and a code* to open the gate—a code of conduct—prevention rather than cure. In the framework of this solution, current successful predatory journals can readily transition to successful non‐predatory journals, whereas new journals will be discouraged from predatory behaviour from the outset.

It is also not the intention of this Opinion to recommend restriction of the diversity of articles, Special Issues, format and policy of journals, which enriches the publication landscape, nor to raise barriers to the creation of new journals, which is essential as knowledge frontiers advance, nor indeed of early career scientists organising and playing a leading role in editing Special Issues, which they should certainly do in journals following good publication practices. Our intention is simply to restrict deviation of journal behaviour and policy from the principles of good science and publication practices.

It is not possible to stop bad behaviour in the world but, in many cases, it is possible to reduce it by restricting the operating space in which it flourishes. Predatory journals thrive on a business model that relies upon unrestricted access to public funds for APCs, ranking by indexing services and a lack of necessity to follow a professional code of conduct, in particular rigorous reviewing coupled with robust editorial decisions. The institution by public funding agencies and indexing services of a requirement for prior accreditation of journals seeking APCs and/or indexing services, based upon a rigorous journal code of conduct, would address all of these issues. This would restrict journal operational space to a degree that is healthy for science, scientists, scientific institutions and science policy. It would reduce wastage of public funds and promote public trust in the science endeavour and appreciation of its value to society.

It is time for researchers worldwide, *the guardians of the science ethos*, to take back science quality control from publishers employing exploitative practices and insist on measures that promote the development of a scientific publication ecosystem of the highest calibre. The consequences of failure to do this are dire. If scientists cannot trust papers, they cannot trust other scientists, so how can politicians and the general public trust scientists? The danger of allowing the present system to continue is that it will lead to decreasing public funding of research, increasing control of scientists by managers who do not understand science and who are unable to judge what is important and achievable, and who will thereby impede rather than promote original research that leads to important new discoveries. Concurrently, there will be an increase in the already heavy burden of bureaucracy that will also both inhibit scientific productivity and discourage the best minds from choosing science as a profession or from remaining in it. And this brings us full circle because, if this happens, funds available for publishing will decrease and the revenues of journals employing predatory practices will decrease. There is thus a motivation for predatory journals to shed exploitational practices, adopt the code of conduct and work for the maintenance of research rigour and the scientific ethos.

## Author Contributions

Kenneth Timmis created an early draft and all authors contributed input that led to the final version.

## Conflicts of Interest

The authors declare no conflicts of interest.

## Data Availability

Data sharing not applicable to this article as no datasets were generated or analysed during the current study.
